# Commentary: Case Report: Hyperbilirubinemia in Gilbert Syndrome Attenuates Covid-19-Induced Metabolic Disturbances

**DOI:** 10.3389/fcvm.2021.685835

**Published:** 2021-07-22

**Authors:** Angelo Minucci, Maria Elisabetta Onori, Andrea Urbani

**Affiliations:** ^1^Molecular and Genomic Diagnostics Unit, Fondazione Policlinico Universitario A. Gemelli IRCCS, Rome, Italy; ^2^Catholic University of the Sacred Heart, Rome, Italy

**Keywords:** bilirubin, COVID-19, Gilbert syndrome, UGT1A1, Crigler Najjar

We have carefully read the recently published article by Al-Kuraishy et al. (1). In this case-report the authors described a patient with Gilbert Syndrome (GS), who presented to the emergency department with fever, dry cough, dyspnea, headache, myalgia, sweating and jaundice and was subsequently diagnosed with COVID-19-induced pneumonia. This patient exhibited a rapid clinical recovery and short hospitalization compared with another COVID-19 positive patient used as control. The authors speculated that hyperbilirubinemia exerted a protective effect in the GS patient due to the known antioxidant, anti-inflammatory and antiviral effects of unconjugated bilirubin. This is the first case in which bilirubin levels are correlated with the prognosis of a patient with COVID-19 infection.

However, based on the reported patient characteristics, we challenge the diagnosis of GS in this patient. This patient presented at time of admission a total serum bilirubin (TSB) level of 6.8 mg/dl with an unconjugated bilirubin level of 6.0, while at time of discharge his TSB had dropped to 3.4 mg/dl. Therefore, in our opinion, the reported TSB does not appear to be related to GS.

In GS patients, the TSB is usually below 3 mg/dl with <20% conjugated bilirubin. Only when associated with other pathological conditions, which increase hemolysis, TSB can be higher, but even then levels are usually below 6 mg/dl (2).

The most common genotype of GS is the homozygous polymorphism *A(TA)7TAA* in the promoter of the UDP-glucuronosyltransferase 1A1 *(UGT1A1)* gene (3). The extra bases reduce the affinity of the binding protein to the TATAA box causing reduced gene expression, which results in a 10% to 35% UGT1A1 enzyme activity reduction. However, more than 130 different pathogenic variants (PVs) in the *UGT1A1* gene are reported (4) and several PVs cause mild UGT activity reduction, which is consistent with GS. Conversely, intermediate TSB, consistent with other rarer forms of hereditary unconjugated hyperbilirubinemia, such as type II Crigler-Najjar syndrome (CNS-2: TSB: 5–20 mg/dl), is observed when the normal allele of a heterozygote CNS-2 PV carrier contains the Gilbert-polymorphism *A(TA)7TAA* (5).

We are aware that distinguishing GS from CNS-2 is often difficult, because many patients show intermediate TSB levels between the defined GS and CNS-2 cut-offs, which complicate a definitive diagnosis in these patients. In these cases, as in the Al-Kuraishy's patient, only genetic testing and clinical information of family members can help to discriminate the two syndromes from each other. In light of these evidences and considering TSB levels, we believe that this patient cannot be diagnosed as GS or CNS-2 according to the existing data, because the TSB level of the patient in the late stage dropped to 3.4 mg/dL.

In addition, the available evidence is insufficient to determine whether TSB level (6.8 mg/dL) is related or unrelated to COVID-19 infection, although a recent systematic review and meta-analysis highlighted that COVID-19 associated liver injury is generally mild and liver injuries are more common in patients with severe COVID-19.

Therefore, in the absence of other clinical evidences, the TSB level of 6.8 mg/dl in the patient described by Al-Kuraishy et al. could be related to genetic alterations of the *UGT1A1* gene and this value cannot be traced back to the presence of GS. However, we underline that the episodes of hyperbilirubinemia in GS patients can be triggered by several factors such as fasting, dehydration, inter-current illnesses, overexertion, and stress. Reducing the total calorie intake, these patients can have a rise up to three times their normal plasma bilirubin concentration within 48 h. The plasma bilirubin returns to normal levels within 24 h with a normal diet. We do not exclude that the TSB trend in this patient may be due to these factors. Unfortunately, in Al-Kuraishy's case report, no clinical information of the patient before admission to the emergency department was reported to justify the bilirubin value of 6.8 mg/dl.

In conclusion, we agree that the study of Al-Kuraishy et al. is particularly relevant regarding the antioxidant role of hyperbilirubinemia in coronavirus disease patients, as already reported by Liu et al. (6). Its beneficial role has been additionally highlighted by Khurana et al., who postulated the use of intravenous administration or inhalational delivery of bilirubin nanomedicine to combat systemic dysfunctions associated with COVID-19 (7). However, we believe that these evidences might involve CNS-2 patients, who are rarer than GS patients; in consequence, it is risky to consider this case as a GS patient based on bilirubin levels, which are apparently not compatible with a GS diagnosis. In our opinion it would have been more correct to describe the case report as “*COVID-19 patient with hereditary unconjugated hyperbilirubinemia.”*

## Author Contributions

AM contributed to conception, design and draft the paper, and agreed to act as guarantor of this paper. AU contributed to draft the article and agreed to act as guarantor of this paper. MEO contributed to draft the paper and agreed to act as guarantor of this paper. All authors contributed to the article and approved the submitted version.

## Conflict of Interest

The authors declare that the research was conducted in the absence of any commercial or financial relationships that could be construed as a potential conflict of interest.

## Abbreviations

GS: Gilbert Syndrome
TSB: total serum bilirubin
UGT1A1: UDP-glucuronosyltransferase 1A1
PVs: pathogenic variants
CNS-2: Crigler–Najjar syndrome type II.
